# Antifungal activity of propolis against Candida spp.

**DOI:** 10.1016/j.nmni.2026.101856

**Published:** 2026-09-23

**Authors:** Etin Rohmatin, Muhamad Sahlan, Heri Hermansyah

**Affiliations:** a Department of Midwifery, Health Polytechnic of the Ministry of Health Tasikmalaya, Tasikmalaya, Indonesia; b Department of Chemical Engineering, Faculty of Engineering, Universitas Indonesia, Depok, Indonesia; c Research Center for Biomedical Engineering, Faculty of Engineering, Universitas Indonesia, Depok, Indonesia; d Department of Interdisciplinary Engineering, Faculty of Engineering, Universitas Indonesia, Depok, Indonesia

**Keywords:** Propolis1, Candida spp.2, Antifungal activity3

## Abstract

Fungal infections caused by Candida species remain a major clinical and pharmacological challenge, ranging from superficial mucocutaneous disease to invasive infections associated with considerable morbidity. Vulvovaginal candidiasis is among the most common manifestations and is frequently recurrent. Increasing antifungal resistance has reduced the effectiveness of azoles and other standard therapies, highlighting the need for alternative or complementary treatment strategies. This systematic review aimed to synthesize recent evidence on the antifungal activity of propolis and its mechanisms of action against Candida species. A systematic literature search was conducted in Scopus and ScienceDirect in accordance with PRISMA 2020 guidelines. Original English-language studies published between 2015 and 2025 were included. Evidence from in vitro, in vivo, and clinical studies evaluating antifungal activity, biofilm inhibition, and mechanistic outcomes was synthesized. **The selection criteria for Candida isolates in the reviewed studies were categorized into two primary groups to ensure both scientific rigor and clinical relevance. Firstly, standard reference strains *C. albicans* ATCC 90028, MTCC 227 were utilized to provide a baseline for antifungal efficacy and to maintain quality control during susceptibility testing Secondly, clinical isolates were sourced from patients with diverse pathological conditions, such as acute vulvovaginal candidiasis (VVC), recurrent VVC (RVVC), and device-associated infections (e.g., from IUD users. This inclusion of real-world strains is critical as clinical isolates often exhibit high biofilm-forming potential and multidrug resistance, which standard strains may not fully represent**. A total of 62 studies met the inclusion criteria. Propolis showed broad-spectrum antifungal activity against *Candida albicans* and non-albicans species, with inhibitory doses varying by geographic origin and chemical composition. Higher potency was reported for Brazilian, Nigerian, and Moroccan propolis, largely linked to phenolic acids, flavonoids, and benzophenones. Reported mechanisms of action included disruption of fungal cell membranes and cell walls, inhibition and eradication of biofilms, suppression of morphogenetic transitions and virulence-related gene expression, synergistic interactions with conventional antifungal agents, and modulation of host immune responses. Despite the limited number of in vivo and clinical investigations, current findings suggest that propolis can produce therapeutic effects similar to conventional antifungal agents, without raising significant safety concerns.

## 1 Introduction

Fungal infections, especially those brought on by Candida species, which are among the most common opportunistic fungal pathogens in humans, remain a major problem in pharmaceutical and clinical practice. A wide range of infections, from superficial mucocutaneous symptoms to invasive systemic disorders linked to significant morbidity, death, and medical expenses, are caused by Candida spp [1,2]. Despite being commensal organisms in the human microbiota, *Candida albicans* and related species can become pathogenic due to changes in host immunity, dysbiosis, prolonged antibiotic exposure, or medical procedures like catheterization [3].

Vulvovaginal candidiasis (VVC) is one of the most clinically significant signs of Candida infection from a pharmacological standpoint, especially in women who are of reproductive age. According to epidemiological data, 70–75% of women will experience at least one episode of VVC during their lifetime, while 40–50% will suffer from recurrent infections [[4], [5], [6]]. Because of its high relapse rates, decreased response to treatment, and detrimental effects on patient quality of life, recurrent vulvovaginal candidiasis (RVVC) presents ongoing treatment challenges [7,8]. The Sustainable Development Goals and the strategic frameworks of the World Health Organization highlight the tight alignment of these challenges with global goals in sexual and reproductive health [[9], [10], [11]].

Azoles, polyenes, and echinocandins are among the synthetic antifungal drugs that are now used in the pharmacological treatment of candidiasis. Azole antifungals, especially fluconazole, continue to be the cornerstone of treatment for VVC in both topical and oral formulations [12,13]. These medications have significant drawbacks despite being widely used, including hepatotoxicity, systemic side effects, local irritation, drug-drug interactions, and pregnancy contraindications [[14], [15], [16]]. More importantly, the global increase in antifungal resistance is jeopardizing the long-term effectiveness of existing antifungal drugs and has emerged as a major issue for both pharmaceutical development and public health [17,18]. There have been more reports of resistance among Candida species, especially *C. albicans*, *C. glabrata*, and *C. krusei*, which can result in treatment failure, longer therapy, and higher healthcare costs [19,20].

Pharmaceutical research has increasingly looked at natural compounds as supplemental or alternative sources of antifungal medicines in response to these difficulties. Because of their structural diversity, multitarget modes of action, comparatively low toxicity, and ability to work in concert with traditional antifungal medications, natural chemicals are seen as promising [21,22]. Propolis, a resinous substance made by honeybees from plant exudates that they enzymatically modify, has drawn strong interest in pharmaceutical science because of its broad range of bioactive properties, including antimicrobial, anti-inflammatory, antioxidant, immunomodulatory, antiviral, and antifungal effects [23,24]. **While propolis is characterized by chemical complexity and geographical variability, recent advancements in high-resolution mass spectrometry (GC-MS/LC-MS) have enabled the identification of stable bioactive markers, such as Sulawesin A and B in Indonesian propolis, which facilitate the standardization required for pharmaceutical development. Moreover, the clinical burden of superficial candidiasis, affecting hundreds of millions of women worldwide, underscores the urgent need for natural alternatives that can bypass the toxic side effects and resistance patterns of conventional azoles** [25,26]

Flavonoids, phenolic acids, aromatic esters, terpenoids, vitamins, and trace minerals make up propolis's extremely varied and complex chemical makeup. Its phytochemical profile is greatly impacted by botanical origin, geographic location, and environmental circumstances [27,28]. Different varieties of propolis exhibit distinct pharmacological activity and chemical signatures, including Brazilian green, European poplar-type, Cuban red, and Indonesian propolis. Sulawesin A and B are among the distinctive bioactive components of Indonesian propolis, especially from Sulawesi and Belitung, together with a variety of flavonoids and polyphenols that 'enhance its biological potency [29,30]. Research on propolis encompasses laboratory studies (in vitro and in vivo) evaluating its mechanisms of action, minimum inhibitory concentrations (MICs), and biofilm inhibition, providing critical insights into its bioactivity and safety profile [31,32]. Clinical studies in humans have assessed the therapeutic efficacy and safety of propolis in formulations such as mouthwashes, vaginal suppositories, and topical preparations for managing oral candidiasis, vaginal yeast infections, and other Candida-related conditions [33]. However, significant variability in propolis's antifungal activity arises from differences in its chemical composition, extraction methods, formulations, and the Candida Species studied [27]. Variations in study designs, ranging from controlled in vitro experiments to observational and interventional clinical studies, create a diverse evidence base that complicates direct comparisons and broad generalizations. [34]. This systematic review synthesizes evidence from laboratory and human-focused studies (2015–2025) on the antifungal activity of propolis against Candida Species. By classifying propolis constituents based on geographic origin and exploring its mechanisms of action, **The novelty of this review lies in its integrated approach to mapping the unique Indonesian biodiversity markers (Sulawesin A and B) against the rising global challenge of antifungal resistance. While earlier works primarily explored standard ethanolic extracts against *C. albicans*,** [26,35]**, this study provides critical evidence for the total eradication of azole-resistant *C. glabrata* using pH-aligned NaDES delivery systems. By synthesizing evidence across chemical profiling, molecular mechanisms, and human clinical trials, this work establishes a new framework for developing sustainable, non-invasive therapies for vulvovaginal candidiasis.**

The review aims to provide a contemporary understanding of its therapeutic potential. These insights may guide researchers, clinicians, and policymakers in developing effective, natural, and sustainable antifungal strategies.

## 2 Results

This systematic review included 62 eligible studies retrieved from the Scopus and ScienceDirect databases using the PRISMA 2020 framework (Fig. 1). The selected articles, published between 2015 and 2025, investigated the antifungal activity of propolis against Candida species across in vitro, in vivo, and clinical study designs. Data extracted from these studies included geographic source, chemical profile, antifungal performance, proposed mechanisms, and therapeutic relevance.

**Fig. 1:**
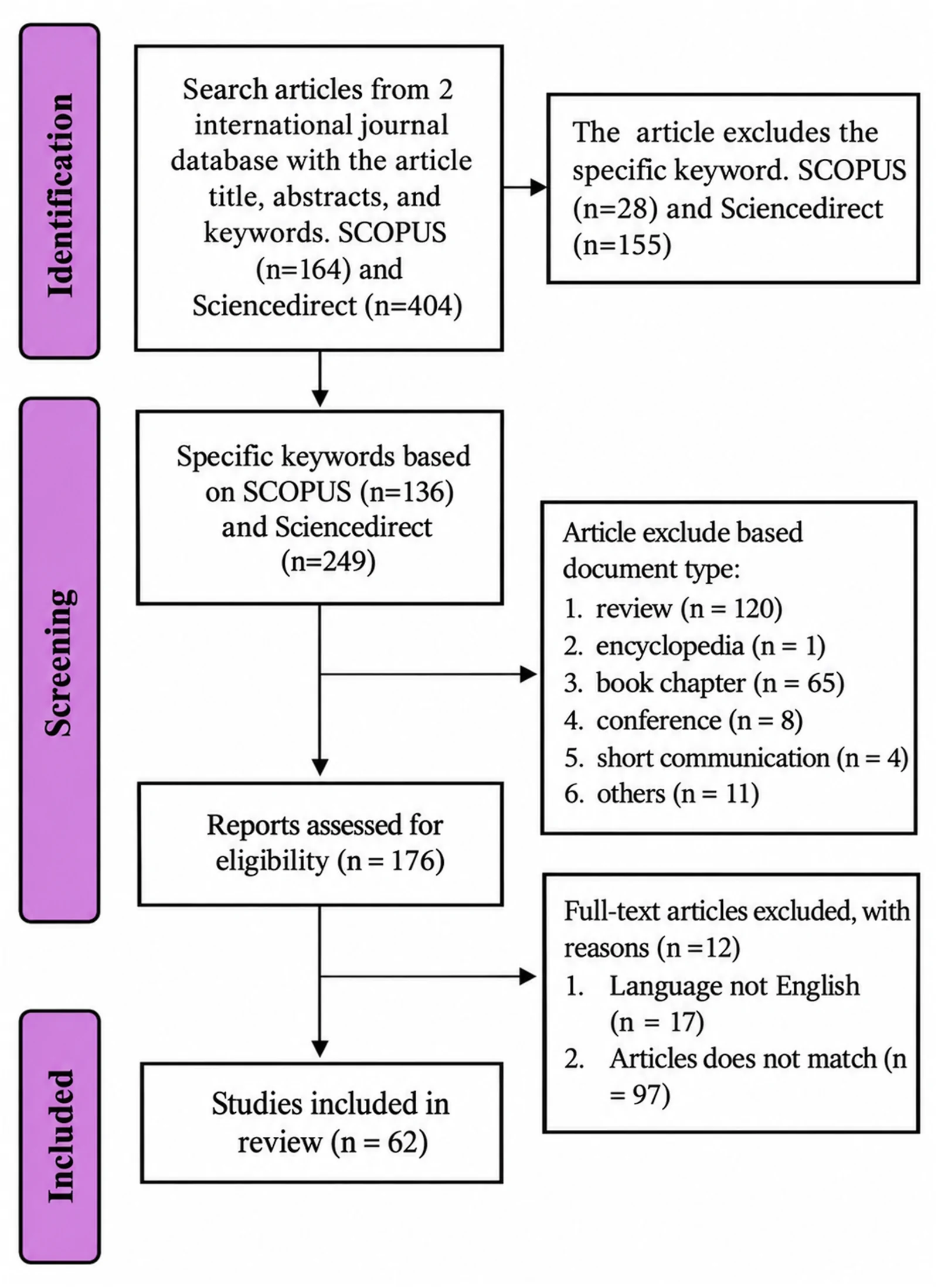
PRISMA 2020 framework.

### 2.1 Characteristics of included studies and antifungal efficacy

Table 1 provides an overview of the features of the included studies. Vulvovaginal candidiasis (VVC), oral candidiasis, denture stomatitis, and device-associated infections were the main topics of in vitro experimental studies, which were followed by in vivo animal models and a small number of human trials. The most frequently investigated species was *Candida albicans*, although some studies also found action against non-albicans Candida species, including *C. glabrata*, *C. krusei*, *C. tropicalis*, *C. parapsilosis*, *C. dubliniensis*, and the developing multidrug-resistant disease Candida auris.

**Table 1:** Antifungal activity of propolis against Candida species: Key bioactive compounds, study design, and therapeutic outcomes.

Country/Region	Author (Year)	Propolis Source/Type	Key Bioactive Compounds Related to Antifungal Activity	Study Design	Dose or Concentration	Comparator	Antifungal Outcomes (MIC/MFC/Biofilm)	Key Findings
**Brazil**	Freires et al., 2016 [36].	Brazilian propolis (types 3 and 13)	p-Coumaric acid, caffeic acid phenethyl ester (CAPE), kaempferol, quercetin, medicarpin, vestitol, formononetin	In vitro: MIC/MFC determination, SEM for biofilm morphology	MIC: 0.2–125 μg/mL; MFC: 125–500 μg/mL; Biofilm disruption: 500 μg/mL	Compared to fungistatic agents (implicit)	Inhibited Candida Spp. (*C. albicans*, *C. dubliniensis*, *C. glabrata*, *C. krusei*, *C. tropicalis*, *C. parapsilosis*); Disrupted preformed/mature biofilms	Types 3 and 13 propolis show potent anti-Candida activity, promising for oral/systemic candidiasis via biofilm disruption
**Brazil**	das Neves et al., 2016 [37].	Brazilian red propolis	Phenolic acids, flavonoids (formononetin, pinocembrin)	In vitro: Bioassay-guided fractionation, MIC determination	MIC: 32–1024 μg/mL (fractions); Formononetin: 25–200 μg/mL	Compared to crude extracts	Formononetin inhibited all Candida strains; Fungicidal against five of six yeasts	Formononetin drives red propolis antifungal activity, supporting therapeutic potential
**Brazil**	Corrêa et al., 2020 [38].	Brazilian propolis	Phenolics (not specified in detail)	In vitro: MIC/MFC, growth kinetics, filamentation, SEM, mutagenicity	Dose-dependent MIC (12 h peak); Biofilm/wall damage: Varied	Compared to conventional antifungals	Inhibited filamentation, caused membrane/wall damage, non-mutagenic	Targets *C. albicans* cell integrity, offering a non-toxic antifungal alternative
**Brazil**	Lima et al., 2022 [39].	Brazilian monofloral honeys (Aroeira, Eucalyptus, Assa-Peixe) with propolis-like properties	Phenolic compounds	In vitro: MIC, biofilm reduction, SEM, Y-H transition, synergism	MIC: 20–40% w/v; Biofilm reduction: 40% w/v	Compared to azoles (miconazole, ketoconazole, itraconazole)	Aroeira honey inhibited planktonic cells, reduced biofilms, reversed azole resistance	Aroeira honey/propolis as a synergistic agent for superficial candidiasis
**Brazil**	Zancanela et al., 2019 [40].	Brazilian propolis (red, green, poplar)	Phenolic acids, flavonoids	In vitro: MIC, SBF release, cytotoxicity, FTIR	MIC: Not quantified; Release: 30–50% (SBF)	Compared to amphotericin B/fluconazole	Red/poplar propolis released active compounds, non-toxic to fibroblasts	NRL-propolis membranes suitable for wound healing with sustained antifungal release
**Brazil**	Sardi et al., 2018 [1].	Caffeic acid derivative esters from propolis	Caffeic acid esters (propyl caffeate)	In vitro: MIC, synergism assay	MIC reduction: Up to 64-fold with fluconazole/nystatin	Compared to fluconazole/nystatin	Synergistic effects, low toxicity to keratinocytes	Propyl caffeate enhances antifungal therapy via synergy and selectivity
**Brazil**	L. F. G. Lima et al., 2024 [41].	Brazilian red propolis	Gutiferone	In vitro/in vivo: MIC, PDT, biomass, mouse model	MIC: 1000 μg/mL; Biofilm reduction: 3.68 Log10 CFU/mL	Compared to fluconazole (implicit)	PDT reduced biofilms/hyphae, lowered tissue CFU	Gutiferone-PDT as a promising alternative for denture stomatitis
**Brazil**	Freitas et al., 2022 [42].	Brazilian red/green propolis	Not specified	In vitro: MIC, adhesion inhibition, in vivo (*T. molitor*)	MIC: 0.03–0.12%	Compared to amphotericin B/fluconazole	Inhibited adhesion (45–82%), synergistic with azoles, protected *T. molitor*	Red propolis effective for denture stomatitis, synergistic with antifungals
**Brazil**	Moita et al., 2022 [43].	Brazilian green propolis (Caatinga)	Fatty acids, triterpenes (cycloartenol, β-amyrin, α-amyrin)	In vitro: MIC/MFC, GC-MS	MIC/MFC: 0.019–3 mg/mL	Compared to none	Inhibited *C. albicans*, *C. tropicalis*, *T. rubrum*; Stable across seasons	Nonpolar fraction drives consistent antifungal activity
**Brazil**	Sokolonski et al., 2021 [44].	Brazilian red/green propolis	Not specified	In vitro: MIC, biofilm inhibition	MIC: Varied (red propolis lower)	Compared to fluconazole/itraconazole	Red propolis inhibited resistant strains, synergistic	Red propolis as auxiliary therapy for denture stomatitis
**Brazil**	Bonfim et al., 2020 [45].	Brazilian propolis	Not specified	In vitro/in vivo: MIC, murine VVC model	Varied (thermoresponsive platform)	Compared to nystatin	Reduced infection in vivo, comparable/better than nystatin	Mucoadhesive propolis systems for VVC treatment
**Brazil**	Martorano-Fernandes et al., 2020 [46].	Brazilian red propolis	Not specified	In vitro: Biofilm inhibition on titanium	MICB50: 15.6 μg/mL; MBEC: 2000 μg/mL	Compared to chlorhexidine/nystatin	Inhibited mono-/co-culture biofilms, reduced metabolism	Red propolis as alternative for peri-implantitis control
**Brazil**	Fasolo et al., 2020 [47].	Brazilian red propolis	Benzophenones	In vitro: MIC, skin/mucosa permeation	MIC: 0.654–2.617 μg/mL	Compared to azoles	High permeation in impaired tissue, cell damage >78%	Nanoemulsions for mucocutaneous fungal infections
**Brazil**	N. C. A. de Lima et al., 2018 [48].	Brazilian green propolis	Not specified	In vitro: Neutrophil oxidative burst	Subinhibitory doses	Compared to RVVC isolates	Increased ROS, enhanced neutrophil killing	Boosts immune response against resistant *C. albicans*
**Brazil**	Teixeira et al., 2025 [49].	Brazilian green propolis	Phenolic acids, flavonoids, coumarins, quinones	In vitro/in vivo: MIC, murine VVC model, UHPLC-MS/MS	MIC: Not specified; 78% encapsulation efficiency	Compared to none	Eliminated *C. albicans* in vivo, no vaginal toxicity	Nano-propolis enhances VVC treatment efficacy
**Brunei**	Zullkiflee et al., 2023 [50].	Bruneian propolis (*H. itama*, *G. thoracica*, *T. binghami*)	Not specified	In vitro: MIC, cytotoxicity, lethality	MIC: 2500–10000 μg/mL	Compared to chlorhexidine	Low antifungal activity, non-toxic	Limited antifungal potential, safe for use
**Bulgaria**	Peycheva et al., 2019 [51].	Bulgarian propolis	Phenolics (Propolin®)	In vivo: Adolescents with gingivitis	10 drops added to toothpaste (20 days)	Compared to marketed toothpaste	Eradicated *C. albicans*, improved gingival health	Enhances oral health, eliminates cariogenic pathogens
**Croatia**	Jug et al., 2017 [52].	Croatian poplar-type propolis	Flavonoids (flavones, flavonols)	In vitro: MIC, extraction optimization	MIC: Varied (80% ethanol optimal)	Compared to water/40% ethanol	Ethanol extracts correlated with flavonoid content, inhibited *C. albicans*	Optimized extraction enhances antifungal efficacy
**Ecuador**	Cabezas-Mera et al., 2023 [53].	Ecuadorian stingless bee propolis	Medioresinol, scopoletin, kaempferol, quercetin, dihydrocaffeic acid	In vitro: MIC, biofilm inhibition, FM/SEM	Biofilm inhibition: 63–80%	Compared to none	Reduced cell count/viability, disrupted biofilm structure	Stingless bee propolis effective against MDR pathogens
**Egypt**	Sawires & Soliman, 2025 [54].	Egyptian propolis	Not specified (FTIR stable)	In vitro: MIC, SEM, irradiation effects	MIC: Varied (1–2 kGy doses)	Compared to antibiotics	Inhibited dermatophytes/*C. albicans*, morphological changes	Irradiated propolis effective for onychomycosis
**Hungary**	Kerek et al., 2023 [55].	Hungarian propolis	Not specified	In vitro: MIC/MEC	MIC: 1.56–400 μg/mL; MEC: 3.125–800 μg/mL	Compared to antibiotics	High activity vs. Gram-positive bacteria/*C. albicans*	Potential for wound infections and trichomoniasis
**Indonesia**	Farida et al., 2020 [29].	Indonesian propolis wax (Tetragonula sp.)	Polyphenols, flavonoids	Clinical: Limited VVC patients	Ovule: Once daily, 7 days	Compared to nystatin	Similar remission, no Candida growth on SDA	Propolis wax as effective VVC therapy alternative
**Indonesia**	Nubla et al., 2024 [56].	Indonesian propolis (Lombok)	Not specified	In vitro: MIC/MEC	MIC: 1.56–400 μg/mL	Compared to standard antifungals	Effective vs. Candida/Cryptococcus	Promising for fungal infections in developing countries
**India**	Shalaby et al., 2024 [57].	Indian propolis	Not specified	In vitro/in vivo: MIC, tissue deposition, murine model	MIC: Varied (nano-formula)	Compared to free propolis	Reduced microbial count, modulated TNF-α/COX-2	Nano-propolis enhances VVC treatment efficacy
**Iran**	Mojgani et al., 2025 [58].	Iranian propolis	Phenolics, flavonoids	In vitro: MIC, biofilm inhibition	MIC: 19.87–150 μg/mL	Compared to antibiotics	High activity vs. *S. mutans*/*C. albicans*, no hemolysis	Potential for oral health applications
**Iran**	Shokri et al., 2017 [59].	Iranian propolis	Phenolic acids (78.2%)	In vitro: MIC/MFC	MIC: 2–20 mg/mL; MFC: 4–25 mg/mL	Compared to azoles	Strong activity vs. *C. krusei* (nail/oral/vaginal isolates)	Promising for cutaneous/mucosal candidiasis
**Iran**	Yazdanian et al., 2022 [60].	Iranian propolis	Thymol, carvacrol, menthol, phenolics	In vitro: MIC, cytotoxicity	MIC: 0.014–0.234 mg/mL (*C. albicans*)	Compared to chlorhexidine	Inhibited oral pathogens, low fibroblast toxicity	Suitable for mouthwashes against dysbiosis
**Iraq**	Al-Oebady, 2025 [61].	Iraqi propolis	Not specified	In vitro: XTT, gene expression (Ece1, Sap5, Als3)	MIC: 40% w/v; MFC: 50% w/v	Compared to untreated biofilms	Reduced biofilm, downregulated virulence genes	Attenuates *C. albicans* virulence via gene suppression
**Malaysia**	Batac et al., 2020 [62].	Malaysian propolis	Not specified	In vitro: MIC, disk diffusion	MIC: Varied (coconut honey lower)	Compared to clotrimazole	Inhibited dermatophytes/*C. albicans*, no zones for some	Propolis/honey for skin fungal infections
**Malaysia**	Shehu et al., 2016 [63].	Malaysian tualang honey/propolis	Phenolic acids (275.6–1754.2 mg/kg), flavonoids	In vitro: MIC/MFC	MIC: 0.375–0.70 mg/mL; MFC: 3.13–12.50 mg/mL	Compared to none	Strong activity vs. *C. albicans*/*C. neoformans*	High phenolics/flavonoids drive antifungal potential
**Mexico**	Ramón-Sierra et al., 2019 [64].	Mexican propolis (*M. beecheii*)	Flavonoids, flavanones–dihydroflavonols	In vitro: MIC/MFC, antioxidant	MIC: 1.62–2.50 μg/mL	Compared to *A. mellifera* propolis	30% lower MIC/MFC than *A. mellifera*	Stingless bee propolis as potent antifungal source
**Morocco**	Aboulghazi et al., 2022 [65].	Moroccan propolis	Epicatechin, apigenin, gallic acid	In vitro: MIC/MFC, antioxidant	MIC: 31.2–62.5 μg/mL; MFC: 62.5–125 μg/mL	Compared to fluconazole	Comparable to fluconazole, high phenolic content	Promising source of bioactive anticandidal compounds
**Nepal**	Okińczyc et al., 2020 [66].	Nepalese propolis (*Apis mellifera*, Trigona sp.)	Flavonoid aglycones, pterocarpans	In vitro: MIC, synergism, filament inhibition	MIC: 50–1000 μg/mL	Compared to antibiotics (amikacin, tetracycline)	Synergistic, inhibited *C. albicans* filamentation	Potent for combination therapy, induces oxidative stress
**Nigeria**	Iyen et al., 2024 [67].	Nigerian red propolis	2′,7-Dihydroxy-3′,4-dimethoxyisoflavan-4-ol (DDI)	In vitro: MIC, molecular docking	MIC: 6.25–25 μg/mL	Compared to fluconazole/amoxicillin	Stronger antifungal than antibacterial	DDI as potential candidiasis drug via enzyme inhibition
**Pitcairn Island**	Georgieva et al., 2018 [68].	Pitcairn Island propolis	Cycloartane triterpenes	In vitro: MIC, MTT assay	MIC: 64 μg/mL (*C. albicans*)	Compared to amphotericin B	High antifungal activity, cytotoxic to cancer cells	Novel triterpenes suggest therapeutic potential
**Poland**	Ożarowski et al., 2022 [69].	Polish propolis	Pinocembrin, pinobanksin-3-acetate, chrysin, galangin	In vitro: MIC/MFC, chemometric analysis	MIC: 32–256 μg/mL; MFC: 64–128 μg/mL	Compared to pure compounds	Mixtures effective, pure compounds inactive	Flavonoid mixtures as artificial propolis for therapy
**Poland**	Gucwa et al., 2018 [70].	Polish propolis (ethanolic extract)	Total polyphenols and flavonoids (individual compounds not isolated mechanistically)	In vitro	Concentration-dependent (tested in μg/mL range); sub-MIC levels used for synergy testing	Fluconazole, itraconazole, voriconazole	MIC determination; checkerboard assay (FICI)	Polish propolis exhibited antifungal activity against *Candida albicans* and non-albicans Candida species; combination with azoles resulted in synergistic or additive effects and reduced azole MIC values in several strains
**Portugal**	A. Bezerra et al., 2024; C. R. F. Bezerra et al., 2020 [71,72].	Portuguese propolis (Montesinho)	H2O2, vanillic acid, pinocembrin	In vitro: MIC, biofilm removal, fluorescence	MIC: 6.75%; Biofilm removal: 10xMIC	Compared to none	Effective vs. MDR biofilms, correlated with H2O2	Chestnut propolis for chronic wound management
**Portugal**	Oliveira et al., 2024 [73].	Portuguese propolis (Gerês)	Not specified	In vitro: MIC, disk diffusion	MIC: Consistent across samples	Compared to erythromycin/vancomycin	Stronger than vancomycin vs. some bacteria	Promising AMR alternative with high antifungal activity
**Romania**	Bostănaru-Iliescu et al., 2025 [74].	Romanian propolis (with nystatin)	Phenolics, flavonoids	In vitro: MIC, SEM, molecular docking	Hydrogel: Varied	Compared to fluconazole-resistant strains	Fungicidal, disrupted membranes	Chitosan-propolis hydrogels for *C. auris* decolonization
**Romania**	Dumitru et al., 2025 [75].	Romanian propolis/honey/royal jelly	Phenolics, AgNPs	In vitro: MIC, spectroscopy	Inhibition zones: 8–10 mm	Compared to antibiotics	Synergistic, low toxicity	Composite films for wound healing/infection control
**Romania**	Vică et al., 2024 [76].	Romanian propolis	Phenols (189.49 mg/kg), flavonoids (88.06 mg/kg)	In vitro: MIC, antioxidant	MIC: Varied (*F. oxysporum* sensitive)	Compared to none	Strong vs. multiple fungi, pollution indicator	Propolis as bioactive phytopharmaceutical
**Turkey**	Arslan et al., 2021 [77].	Turkish propolis (Ardahan/Erzurum)	Not specified	In vitro: MIC, cytotoxicity	MIC: 62.5–125 μg/mL	Compared to none	Anticarcinogenic, antifungal variation by origin	Geographical origin impacts biological activity
**Turkey**	Degirmencioglu et al., 2019 [78].	Turkish propolis (Anatolian)	Quercetin, CAPE, galangin	In vitro: MIC, HPTLC, PCA	MIC: 128–512 μg/mL	Compared to antibiotics	High antioxidant/antimicrobial activity	O-type propolis dominant, new 3MQ-type identified

Propolis exhibited broad-spectrum antifungal activity, with reported minimum inhibitory concentrations (MICs) ranging from as low as 0.2 μg/mL for Brazilian propolis (Freires et al., 2016) to as high as 10,000 μg/mL for Bruneian propolis [50]. Propolis samples originating from regions rich in flavonoids and phenolic compounds particularly Brazil, Nigeria, and Morocco, consistently demonstrated lower MIC values, reflecting greater antifungal potency.

Brazilian propolis, especially the red and green varieties, demonstrated strong antifungal activity against a variety of Candida species, with minimum fungicidal concentrations (MFCs) ranging from 4 to 500 μg/mL and MIC values between 0.2 and 125 μg/mL [36,38,42]. Nigerian red propolis, rich in 2′,7-dihydroxy-3′,4-dimethoxyisoflavan-4-ol (DDI), demonstrated MIC values of 6.25–25 μg/mL, exceeding standard antifungals in various studies [67]. Epicatechin and apigenin-containing Moroccan propolis showed MICs of 31.2–62.5 μg/mL, which is similar to fluconazole [65].

Bruneian propolis, on the other hand, showed comparatively limited antifungal action (MIC 2500–10,000 μg/mL), either as a result of lower quantities or the lack of important bioactive components [50]. Moderate to high antifungal activity was identified in propolis from Poland, Iran, Ecuador, Portugal, Turkey, and Mexico, frequently linked with complex flavonoid mixes rather than isolated compounds [53,69,71].

Findings related to biofilm activity were commonly reported across the included studies. Propolis from Ecuadorian stingless bees was shown to reduce biofilm formation by 63–80%, whereas Brazilian red propolis inhibited biofilm metabolic activity (MICB_50_) at 15.6 μg/mL and achieved biofilm eradication at higher concentrations [46,53].

### 2.2 Mechanisms of antifungal action

The antifungal mechanisms of propolis identified in the included studies are presented in Table 2. Propolis exhibited multifaceted antifungal activity by simultaneously targeting fungal structure and virulence.

**Table 2:** Mechanisms of antifungal action of propolis against Candida species.

Mechanism Category	Proposed Antifungal Mechanism	Candida Species	Reference
**Membrane and Cell Wall Disruption**	Disrupts fungal membrane integrity through lipophilic phenolic compounds (e.g., p-coumaric acid, CAPE) that interact with lipid bilayers, leading to leakage of intracellular components and loss of cellular homeostasis	*C. albicans*	Corrêa et al., 2020 [38].
	Induces marked morphological alterations, including cell shrinkage and membrane rupture, particularly after low-dose irradiation (1–2 kGy), likely mediated by phenolic-associated oxidative damage to lipid membranes as observed by SEM	*C. albicans*, *C. krusei*	Sawires & Soliman, 2025 [54].
	Exhibits potential inhibition of dihydrofolate reductase (DHFR) based on molecular docking analyses, suggesting interference with folate metabolism and cellular viability	*C. auris*	Bostănaru-Iliescu et al., 2025 [74].
	Enhances antifungal efficacy in mucosal tissues via nanoemulsion delivery systems that improve penetration and bioavailability of benzophenone-rich propolis, resulting in extensive fungal cell damage	*C. albicans*	Fasolo et al., 2020 [47].
	Inhibits fungal growth through disruption of membrane integrity, likely mediated by flavonoid and polyphenol interactions with membrane lipids	*C. albicans*	Farida, S. et al., 2020 [29].
**Biofilm Inhibition/Disruption**	Reduces viability of preformed and mature biofilms by interfering with cell adhesion and extracellular matrix integrity, as demonstrated by SEM	*C. albicans*, *C. dubliniensis*, *C. glabrata*, *C. krusei*, *C. tropicalis*, *C. parapsilosis*	Freires et al., 2016 [36].
	Inhibits biofilm formation and yeast-to-hyphae transition by interfering with regulatory pathways involved in matrix production and morphogenesis	*C. albicans*	W. G. Lima et al., 2022 [39].
	Reduces biofilm formation on titanium surfaces by impairing fungal adhesion and metabolic activity, particularly in mono- and mixed-species biofilms	*C. albicans*	Martorano-Fernandes, L. et al., 2020 [46].
	Disrupts established biofilms through photodynamic therapy mediated by gutiferone-induced ROS generation, significantly reducing fungal burden in vivo	*C. albicans*	L. F. G. Lima et al., 2024 [41].
	Reduces the viability and structural integrity of mature nail biofilms, as evidenced by significant reductions in fungal burden and biofilm architecture disruption.	*C. albicans*	Barros et al., 2023 [79].
	Decreases biofilm biomass through combined effects of hydrogen peroxide and phenolic compounds, disrupting matrix components and fungal cells	*C. albicans*	C. R. F. Bezerra et al., 2020 [72].
	Inhibits early biofilm formation by impairing adhesion and extracellular matrix production	*C. albicans*, *C. glabrata*	Cabezas-Mera et al., 2023 [53].
	Significantly reduces fungal adhesion and biofilm establishment in vitro and in vivo models	*C. albicans*	Freitas et al., 2022 [42].
**Gene Expression Suppression**	Downregulates virulence genes (ECE1, SAP5, ALS3) involved in hyphal extension, protease activity, and adhesion, reducing pathogenicity and biofilm formation	*C. albicans*	Al-Oebady, 2025 [61].
**Inhibition of Filamentation**	Suppresses yeast-to-hyphae transition by interfering with cAMP–PKA-associated morphogenetic signaling pathways	*C. albicans*	W. G. Lima et al., 2022 [39].
	Inhibits morphogenesis via oxidative stress induction and modulation of filamentation-related transcriptional regulators	*C. albicans*	Okińczyc et al., 2020 [66].
	Primarily impairs virulence-associated stress adaptation and cellular homeostasis in non-hyphal Candida species rather than targeting filamentation-related mechanisms.	*C. glabrata*	Argüelles et al., 2021, 2023 [80,81].
**Synergism with Antifungals**	Enhances azole and polyene activity, resulting in significant MIC reduction, potentially through increased membrane susceptibility	*C. albicans*	Sardi et al., 2018 [1].
	Restores azole susceptibility in resistant strains, with findings suggesting involvement of efflux-related mechanisms	*C. albicans*	W. G. Lima et al., 2022 [39].
	Acts synergistically with fluconazole and itraconazole, likely by facilitating antifungal penetration through membrane destabilization	*C. albicans*	Sokolonski et al., 2021 [44].
	Improves antifungal penetration and activity within biofilms when combined with azoles	*C. albicans*, *C. parapsilosis*	Bergamini et al., 2025 [82].
	Acts synergistically with fluconazole through propolis-derived compounds (notably CAPE and quercetin), restoring azole susceptibility in resistant strains by reducing fungal burden, impairing biofilm formation, and suppressing virulence-associated gene expression.	*C. albicans*	Gao et al., 2016; Sun et al., 2018 [83,84].
**Immune Modulation**	Stimulates neutrophil oxidative burst and ROS production, enhancing immune-mediated fungal killing.	*C. albicans*	N. C. A. de Lima et al., 2018 [48].
**Enzyme Inhibition**	Shows potential inhibition of lanosterol 14α-demethylase based on in silico molecular docking, suggesting interference with ergosterol biosynthesis.	*C. albicans*	Iyen et al., 2024 [67].

### 2.3 Structural and functional cell disruption

Membrane and cell wall disruption was the most often observed mechanism. P-coumaric acid, CAPE, pinocembrin, quercetin, and benzophenones are examples of phenolic compounds and flavonoids that have been demonstrated to integrate into fungal lipid bilayers, resulting in pore formation, membrane destabilization, and cytoplasmic leakage, which ultimately cause fungal cell death [38,47]. Electron microscopy confirmed cell shrinkage, wall breakage, and loss of membrane integrity in *C. albicans* and *C. krusei* [54].

In silico analyses and molecular docking studies have further shown that compounds in propolis can interact with key fungal enzymes, such as lanosterol 14α-demethylase (ERG11) and dihydrofolate reductase (DHFR), thereby interfering with ergosterol biosynthesis and membrane formation [67,74]. Synergistic effects were also reported when propolis was combined with chelating agents or standard antifungals, resulting in greater membrane disruption, increased permeability, and more rapid fungal cell death [85].

### 2.4 Biofilm disruption and morphogenesis inhibition

Inhibition and eradication of biofilms represent a major antifungal mechanism of propolis. Studies showed that propolis disrupts both developing and mature biofilms by impairing cell adhesion, extracellular matrix formation, and metabolic activity [36,46]. Treatment with propolis resulted in reductions of up to 4 log CFU in mature *C. albicans* biofilms [79].

Propolis also inhibited the yeast-to-hyphae transition by modulating cAMP–PKA signaling and inducing oxidative stress, resulting in mitochondrial dysfunction and apoptosis [35,39,66].

### 2.5 Molecular modulation, synergism, and immune enhancement

At the molecular level, propolis suppressed the expression of virulence-associated genes such as Ece1, Sap5, and Als3, leading to reduced adhesion, filamentation, and pathogenic potential [61]. Strong synergistic interactions with azoles and polyenes were observed, producing up to 64-fold decreases in MICs, reversing antifungal resistance, and improving activity against biofilm-related infections [1,86].

Propolis also enhanced host immune responses by promoting neutrophil oxidative burst and ROS production, increasing fungal clearance, particularly in resistant *C. albicans* strains [48].

Geographic origin played a key role in determining the antifungal effectiveness of propolis, likely due to regional variation in botanical sources and chemical composition. Brazilian propolis consistently demonstrated the strongest antifungal activity, with Nigerian and Moroccan propolis showing comparable but slightly lower potency. Propolis from Europe and Asia tended to exhibit moderate effects, while samples from Brunei showed limited antifungal activity.

Clinical and in vivo findings reinforced the antifungal potential of propolis previously demonstrated in vitro. Propolis wax ovules derived from Tetragonula sp. achieved clinical remission rates in vulvovaginal candidiasis comparable to those of nystatin, without additional adverse effects [29]. Similar therapeutic outcomes were reported when propolis was compared with fluconazole in other VVC studies [26]. In pediatric oral candidiasis, propolis yielded a mean inhibition zone of 9.5 mm, indicating clinically relevant antifungal activity and good tolerability [87].

## 3 Discussion

**Recent advancements in antifungal research have introduced several novel compounds. For instance, Juglone (5-hydroxy-1,4-naphthoquinone) has demonstrated robust in vitro activity against both fluconazole-resistant and sensitive Candida isolates by inducing oxidative damage, a mechanism that mirrors the action of phenolic compounds found in propolis** [24]**. Similarly, the newly developed azole analogs ATTAF-1 and ATTAF-2 show promise in overcoming resistance; however, propolis offers a distinct advantage as a natural alternative with lower host-cell toxicity (high CC50 values), making it potentially safer for prolonged mucosal applications** [24,25]**, concern regarding weak activity is addressed by the potency of propolis against multidrug-resistant (MDR) strains. In specific assays, propolis extracts achieved complete eradication (0 CFU/ml) of *C. glabrata*, outperforming standard Fluconazole. The significance of propolis lies not in a single magic bullet compound, but in its multi-targeted mechanism simultaneously disrupting biofilm architecture, inhibiting the yeast-to-hyphae transition, and inducing apoptosis which significantly reduces the likelihood of fungi developing compensatory resistance mechanisms.**

### 3.1 Structural and functional disruption of fungal cells

The antifungal activity of propolis is strongly associated with its ability to compromise the structural and functional integrity of fungal cells, particularly the cell membrane and cell wall. Rather than just preventing fungal growth, propolis exhibits fungicidal properties by targeting key components essential for cellular homeostasis. The phenolic acids and flavonoids contained in propolis display amphipathic properties that allow them to interact with fungal phospholipid bilayers, leading to increased membrane permeability and collapse of electrochemical gradients. This approach is similar to membrane-targeting antifungals, but it is based on a complex mixture of naturally occurring chemicals rather than a single active molecule.

Beyond direct physical disruption, accumulating data suggests that propolis interacts with internal metabolic processes required for membrane production and maintenance. Flavonoids and similar phenolics may interact with major fungal enzymes involved in sterol and folate metabolism, such as dihydrofolate reductase and lanosterol 14α-demethylase (ERG11) [74,88]. These interactions suggest that propolis has antifungal properties not just at the membrane level, but also via upstream metabolic processes that are crucial for fungal viability. Such dual targeting may help to limit the possibility of resistance development, as disrupting both structural and metabolic processes simultaneously provides a significant adaptive challenge for Candida species.

The antifungal efficacy of propolis can be further improved through formulation approaches and the use of external modulators. Nanoemulsion-based delivery systems have been shown to enhance the tissue penetration and bioavailability of lipophilic constituents, thereby intensifying membrane disruption in mucocutaneous tissues [47]. Yeniçeri Özata et al. (2022) found that combining propolis extracts with chelating agents like EDTA or phytic acid weakens ionic stability of fungal membranes, exacerbating cellular damage. These findings highlight propolis as a multifunctional antifungal agent capable of inducing both physical and biochemical stress in fungal cells [85]. **The differential response between fluconazole-sensitive and fluconazole-resistant isolates highlights the unique pharmacological profile of propolis. For sensitive isolates, propolis often acts synergistically with conventional azoles, significantly reducing the Minimum Inhibitory Concentration (MIC) of fluconazole sometimes up to 64-fold thereby suggesting its role as a potent adjuvant to prevent the emergence of drug resistance** [29,35]**. Conversely, in resistant isolates such as *C. glabrata* or *C. krusei*, propolis bypasses traditional resistance mechanisms (like ERG11 mutations or efflux pump overexpression) through a 'multi-target' strategy** [29,35]**. This involves the simultaneous disruption of cell membrane integrity, inhibition of the yeast-to-hyphal transition (germ tube formation), and induction of oxidative stress via Reactive Oxygen Species (ROS) production, which collectively lead to fungal cell death even in strains where fluconazole fails** [20,24,29]

### 3.2 Disruption of biofilm formation and morphogenesis

Biofilm formation and morphological switching are central virulence strategies of Candida species, underpinning chronic infection, immune evasion, and antifungal resistance. The ability of propolis to target these processes highlights its potential value in treating recalcitrant fungal infections. Rather than targeting planktonic cells, propolis damages the structural integrity of established biofilms and interferes with early adhesion and extracellular matrix (ECM) synthesis, reducing biofilm cohesiveness and durability.

The ability of propolis to disrupt biofilms appears to be linked to its capacity to destabilize key components of the extracellular matrix (ECM), including polysaccharides, proteins, and extracellular DNA, which are critical for biofilm stability and antifungal tolerance [89]. Propolis makes fungal cells more susceptible to both host immune responses and co-administered antifungal medicines by lowering ECM density and integrity. This mechanism is especially important in device-associated infections, where biofilm formation on dentures, implants, and medical surfaces poses a significant clinical problem [46,90].

Propolis also affects Candida morphogenesis by inhibiting the yeast-to-hyphae transition, a process that is critical for tissue invasion and pathogenicity. Flavonoids and phenolic constituents of propolis interfere with intracellular signaling pathways, including the cAMP–PKA axis and transcriptional regulators involved in filamentation [66]. Propolis-induced oxidative stress further promotes mitochondrial dysfunction and activation of apoptotic pathways, thereby restricting hyphal formation and virulence. The simultaneous inhibition of biofilm development and morphological switching highlights propolis as an anti-virulence agent rather than merely a fungistatic compound.

### 3.3 Clinical and in vivo relevance

Growing clinical and in vivo evidence highlights the translational potential of propolis, demonstrating both its safety and effectiveness. Clinical studies in oral and vaginal candidiasis have shown that propolis-based formulations can produce outcomes comparable to those of standard antifungal agents, including nystatin and fluconazole, without an associated increase in adverse effects [29,51]. These observations are particularly important given the rising incidence of antifungal resistance and the limited tolerability of some synthetic treatments.

Animal studies have provided additional support for the antifungal activity of propolis in physiologically relevant models. Propolis formulations, including thermoresponsive and nano-encapsulated systems, have shown excellent fungal clearance with no visible tissue toxicity [45,49]. These findings highlight how formulation optimization can play a critical role in enhancing therapeutic outcomes.

Propolis also exhibits strong synergistic effects when combined with standard antifungal drugs. By increasing membrane permeability and suppressing efflux pump function, propolis enhances antifungal drug uptake and can restore susceptibility in resistant Candida strains [1,44].

This synergy supports the use of propolis as an adjuvant therapy, potentially lowering antifungal dosages and preventing resistance development. Taken together, these findings suggest that propolis is a viable complimentary drug with multiple mechanisms that can be used to treat infection and manage resistance.

### 3.4 Integrative perspective on antifungal mechanisms

Based on the integrated findings, the antifungal activity of propolis against Candida species can be grouped into three interrelated mechanisms [1]: disruption of fungal cell structure and function [2], suppression of biofilm development and morphological transitions, and [3] modulation of molecular and genetic pathways linked to virulence and antifungal resistance. The confluence of these processes shows propolis' complex phytochemical composition and ability to exert antifungal actions across numerous biological levels. Such multimodal activity not only improves therapeutic efficacy but also provides a strategic advantage in addressing the developing problem of antifungal resistance.

## 4 Conclusion

In conclusion, propolis demonstrates substantial antifungal potential, particularly against resistant Candida species. However, it is essential to remain consistent. Propolis is currently most appropriately positioned as a complementary therapeutic agent or a bioactive component in innovative drug delivery systems, this review provides a critical reference for antifungal drug research by mapping the shift from traditional ethanol extraction to synergistic, pH-aligned delivery systems like NaDES and microcapsules, but rather than a complete replacement for standard antifungal medications [29,91]. Further clinical research is required to fully validate its efficacy and safety in human subjects. By bridging the gap between molecular mechanisms and human clinical remission, this work establishes that propolis is not merely a traditional remedy but a scientifically valid precursor for the next generation of sustainable antifungal therapeutics.

## 5 Study limitations

Despite the promising findings, this review acknowledges several limitations. First, the chemical variability of propolis due to its diverse geographical origins (e.g., Sulawesi vs. Chihuahua) remains a major hurdle for universal dose standardization [24,29]. Second, there is a significant 'in vitro to in vivo gap laboratory results, such as inhibition halos or MICs, do not always correlate perfectly with clinical outcomes due to the complexity of the vaginal environment and the phenomenon of 'trailing growth' in certain Candida species [26]. Lastly, there is a relative scarcity of large-scale randomized controlled trials (RCTs) compared to laboratory-based studies, which limits the ability to generalize these results to broader clinical populations [26].

## CRediT authorship contribution statement

**Etin Rohmatin:** Writing – original draft, Conceptualization, Methodology, Data curation. **Muhamad Sahlan:** Writing – review & editing, Funding acquisition, Project administration, Validation. **Heri Hermansyah:** Conceptualization, Project administration.

## Declaration of competing interest

The authors declare that they have no known competing financial interests or personal relationships that could have appeared to influence the work reported in this paper.
